# COVID-19 impact on labour relations in Palestine, the need for legal reform

**DOI:** 10.1016/j.heliyon.2021.e08313

**Published:** 2021-11-04

**Authors:** Mahmoud Fayyad, Abdel Raouf Al-Sinnawi

**Affiliations:** a College of Law, University of Sharjah, United Arab Emirates; b College of Law, Al-Quds University, Palestine

**Keywords:** COVID-19 and labour market, Labour market in the West Bank, Labour market in Palestine

## Abstract

**Objective:**

This research aims to examine how lawful the measures taken by employers against workers are in the context of the COVID-19 pandemic, and to assess performance of the Ministry of Labour (MoL) and various trade unions in representing workers’ interests.

**Methodology:**

After an intensive literature review on the subject, 12 cases – representing different areas and economic sectors in the West Bank – were investigated. Then, free discussions were conducted with a focus group of 24 stakeholders (employers, employees, MoL representatives, trade union representatives, businessmen association representatives, lawyers, judges, and academics) to explore decisions made against employees, examine how lawful these decisions are, and present the evaluation of these groups to the MoL and trade unions. The discussions were followed by a quantitative questionnaire for 297 employees who were affected by unfair decisions and 87 employers representing different areas and sectors in the West Bank. A brainstorming session, involving two judges, two lawyers and four legal academics, was held to discuss research outcomes and benefit from their feedback and recommendations.

**Research problem:**

To demonstrate how lawful the measures taken by employers against workers are in the context of the COVID-19 pandemic, and to assess performance of the MoL and various trade unions in representing workers’ interests.

**Findings:**

The research paper reveals the illegality of the measures taken against employees in the context of responding to the economic effects of the pandemic. It reflects clear and significant dissatisfaction with the decisions made by trade unions and the MoL. The paper also recommends that some amendments need to be introduced to the Palestinian Labour Law.

**Originality/value:**

This research paper pursues the illegality of decisions made by employers against workers in the context of addressing the economic losses caused by the pandemic. It concludes that ambiguous provisions of the Palestinian Labour Law were a main factor that allowed employers to abuse these decisions. The tripartite agreement organised by the MoL was against workers interest and gave rise to many legal and economic challenges. The need to review the structure and functioning of trade unions in the West Bank is highlighted in this research paper.

## 1 Introduction

**Figure 1:**
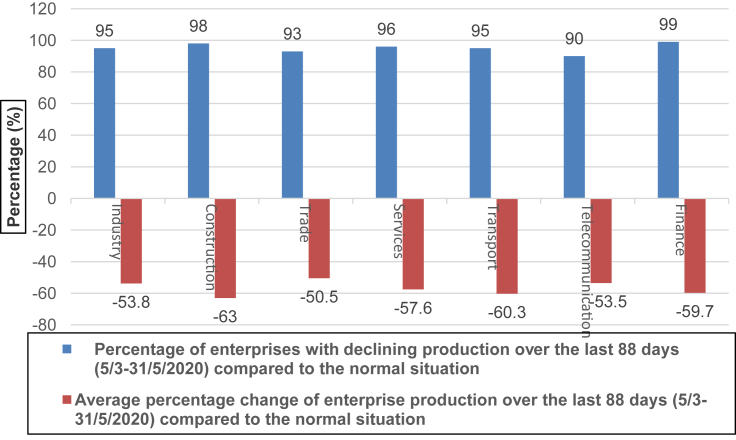
Indicators of declining economic sectors. Source: Palestinian Central Bureau of Statistics, Press Report on Economic Forecasts for 2021, The Performance of the Palestinian Economy during 2020 dated 20/12/2020.

The coronavirus (COVID-19) pandemic has spread around the world (Baek et al., 2020; Deakin and Meng, 2020; Forsythe et al., 2020; Webb Hooper et al., 2020). In addition to a large number of fatalities and huge suffering (Gozgor, 2021; Zhang et al., 2021), it has had a significant negative economic impact (Coibion et al., 2020; Cowan, 2020; Yearby and Mohapatra, 2020); millions of employees around the world are suffering financially due to job loss or unfair measures taken by employers (Alderman et al., 2020; L. P. Beland et al., 2020, Beland et al., 2020; Radu, 2020). In Palestine, namely the West Bank, due to the outbreak of the COVID-19 pandemic and failure of the health sector to respond to widespread infections, most West Bank-based public and private entities have been effectively under intermittent lockdown since the second half of March 2020. The Palestinian economy has recorded a general decline of 12% in 2020. More than two thirds of enterprises were closed during lockdown period (5 March-31 May 2020). Sales/production volume have decreased by 94% during the three-month lockdown, with the average sales/production dropping by 53% compared to normal situation. Establishments operating in construction sector have recorded the highest decline of the average sales/production (63%), followed by the transportation sector (60%) (See Figure 1 below).

In response to the COVID-19 pandemic, formal statistics showed that 14% of enterprises dismissed their employees, 8% reduced wages of their employees, 11% forced their workers to take unpaid leaves, and 9% obliged them to get paid annual leaves. In general, some 66,000 employees lost their jobs during 2020. Unemployment recorded an unprecedented rate (27.8%), The per capita GDP has decreased by 14%. The total number of employees dropped by 78,000, while the number of workers in Israel declined by around 34,000. The decrease was caused by the preventative measures taken to limit the outbreak of COVID-19. However, as those measures were recently eased and work was gradually resumed, the number of employees in the Palestinian domestic labour market increased by 12,000 during the third quarter of 2020 compared to the previous quarter. The number of Palestinians employed in Israel and Israeli settlements rose by some 33,000.

Less than two months later, the lockdown was completely lifted. However, workers were surprised by many individual decisions made by employers, allegedly in response to the economic impact of the pandemic. Among these, employers terminated employment contracts, forced workers to take unpaid leaves, and modified employment conditions. These actions were justified by “restructuring enterprises in view of the financial losses caused by the pandemic.” This research paper aims to demonstrate how lawful the measures taken by employers against workers are in the context of the COVID-19 pandemic, and to assess performance of the MoL and various trade unions in representing workers’ interests.

### 1.1 Novelty of the study

This is the first academic research which examines stakeholders' satisfaction with the procedures and measures taken by trade unions and the MoL in response to the COVID-19 pandemic impacts and to protect workers’ interests from decisions made by employers. It is also the first study to assess how lawful these decisions and the tripartite agreement were in the light of national legislation and case law of Palestinian high courts. The study provides a review of the Islamic *Fiqh* [jurisprudence] as a main source of legislation in Palestine. It is also the first study which uses a combination of quantitative and qualitative research methods in this field.

## 2 Methodology

To achieve objectives of the study, research steps were as follows:

1. Desk research took place, including a literature review.
2. Twelve labour cases, on which unfair decisions were rendered against workers due to the COVID-19 pandemic in different areas and economic sectors in the West Bank, were investigated. Generally, the discussion aimed to identify the measures taken against employees in the context of the pandemic. It also sought to evaluate trade unions and MoL performance.
3. Free discussions (face-to-face interviews) were held with a focus group of 24 stakeholders (employers, employees, MoL representatives, trade union representatives, businessmen associations representatives, lawyers, judges, and academics) to discuss decisions made against employees, examine how lawful these decisions were, and evaluate trade unions and MoL practice.
4. A group of 12 volunteer field researchers conducted face-to-face and WhatsApp interviews with a random sample of 1,000 workers across the West Bank to detect those groups affected by unfair decisions (297 employees, representing 29.7 percent of the entire sample, were listed). To receive their feedback on the research questions, quantitative questionnaires were prepared and distributed to this group (297 employees).
5. One hundred employers, representing different areas and sectors in the West Bank, were asked to answer another questionnaire. Of these, 87 employers (87 percent) responded the questionnaire.
6. The results of the questionnaires were compared, discussed, and reported in a first draft.
7. The first draft was presented and discussed in a brainstorming session (two judges, two lawyers and four legal academics) to be informed by their feedback and recommendations.
8. A conclusion and recommendations were added.

### 2.1 Limitations of the study

Due to the lack of financial support for researchers and movement restrictions throughout the occupied Palestinian territories, the scope of geographical research was limited to the West Bank and excluded the Gaza Strip and East Jerusalem. Due to the limited financial support, the number of recruited participants was relatively low. Researchers relied on volunteer research assistants to complete the field research and organise interviews. Also, interviews with representatives of the executive authority only involved those of the MoL. Because their requests were declined, researchers could not conduct interviews with representatives of other sectors, such as governorates and the Ministry of National Economy.

## 3 Lawfulness of employer decisions against workers

As shown in Table 1 below, the field research has shown many unfair decisions made by employers against workers. These decisions were made as soon as enterprises were reopened, workers resumed their work, enterprise business returned to normalcy. In some cases, many employment contracts dated back to 10 years or more the field research has shown that a significant number of workers, against whom unfair decisions were made, had been employed at enterprises, which were never affected by the pandemic. These included the education sector 20% and civil society organisations 11%, which maintained their business by telecommuting. Many cases demonstrated that employers carried out unprofessional practices in dismissal decisions; workers were falsely informed that such decisions are lawful and are issued according to the recommendations of legal advisors. Ostensibly, some employers deliberately laid off many workers and minimised enterprise activity. Only a few workers with high salaries were dismissed. In one case, an enterprise owner reported to workers that he was about to sack 80 out of 120 workers. Still, the employer's decision only affected a few long-serving and highly paid workers.

**Table 1:** Information for questionnaired employees and employers.

**Employee's Gender (297 Respondents)**	
Male	220 (74%)
Female	77 (26%)
**Employee's Age (297 Respondents)**	
18–30 Years	113 (38%)
30–40 Years	127 (43%)
40-50- Years	51 (17%)
More than 50 years	6 (2%)
**Employer's Decision (297 Respondents)**	
Contract Termination	50 (17%)
Unpaid Leave	99 (37%)
Wage decrease	57 (19%)
Change wage calculation method	53 (18%)
Change notion of work	33 (12%)
**Nature of the Firm's activity (297 Respondents)**	
Education	59 (20%)
Factory	56 (19%)
Company	41 (14%)
Hotel	39 (13%)
Restaurant	20 (7%)
**History of Employee’ Contract (297 Respondents)**	
Less than 5 years	119 (40%)
5–10 Years	135 (32%)
10–15 Years	47 (16%)
15–20 Years	25 (8%)
More than 20 Years	9 (3%)
**Firm’ History (87 Respondents)**	
Less than 5 years	33 (42%)
5–10 years	17 (22%)
10–15 years	13 (17%)
More than 15 years	15 (19%)
**Employee’ Monthly Wage (297 Respondents)**	
Less than 2000 NIS	71 (24%)
2000-4000 NIS	135 (46%)
4000-6000 NIS	62 (21%)
More than 6000 NIS	26 (9%)
**Firm's number of Employees (87 Respondents)**	
Less than 5 Employees	38 (49%)
5-10 Employees	16 (20%)
10-20 Employees	8 (10%)
More than 20 Employees	16 (20%)
**Firm's Level of Loss (87 Respondents)**	
Grave Loss	30 (38.5%)
Medium Loss	34 (43.6%)
Little Loss	11 (14.1%)
There is no loss	3 (3.8%)

### 3.1 Terminating employment contracts on the pretext of enterprise restructuring

The field research has shown that many economic sectors have been significantly influenced by the pandemic, but COVID-19 did not affect all the enterprises which took arbitrary action against relevant workers (See Figure 1). For example, the field research detected private schools’ decisions, which have been operated since the 1990s, against teachers although those schools continued working online and tuition fees were not affected as the greatest portion of fees had already been paid before the pandemic. In another case, a dismissed employee said: “Hotel profits over the past two years shows huge profits. Contrary to their claim, there is no need for restructuring.” Similarly, a worker reported that an enterprise was affected by the pandemic, but business profits made over 20 years cover these losses and more. Table 1 above shows the history of enterprises which took measures against workers. It demonstrates that 46% of these enterprises have been doing business for over 10 years.

Most of the employers invoked the provisions of Article 41 of the Labour Law to terminate workers contracts. Article 41 authorises the employer to terminate a labour contract for technical reasons or due to financial loss, which makes it necessary to reduce the number of workers. In such a case, workers will maintain their right to an allowance in lieu of notice and severance pay (Bell et al., 2020), provided that the Ministry is duly notified thereof.^1^ The Jerusalem Court of Appeals upheld that the termination of an employment contract due to the financial distress of an enterprise, which requires that the number of workers be reduced and makes it incapable of debt repayment, is deemed to be a lawful cause to terminate the contracts of some workers for the purposes of downsizing enterprise business.^2^ The Court also considered that an employment contract is terminated *de jure* in the event of closure under a court or administrative decision for a period of over two months. In such a case, the employer is obliged to reimburse all worker benefits, with the exception of unfair dismissal compensation.^3^ Employers carry the onus of proof to fulfil the legally prescribed terms and conditions for using their right to terminate the employment contracts of some workers for the purpose of enterprise restructuring.^4^ By contrast, the Court of Cassation ruled for obliging employers to pay unfair dismissal compensation to workers in cases where an employment contract is terminated in contravention to the provisions of the law.^5^

Additionally, the field research has also found out that dismissal decisions were, in many cases, limited to workers and employees, who exercised the workers’ right to protest. For instance, ten workers were laid off from an enterprise because they had engaged in a sit-in protest against arbitrary decisions made by employers. Dismissal did not affect other workers. On another occasion, an enterprise management took advantage of the COVID-19 outbreak to terminate the employment contract of only one worker. It did not pay unfair dismissal compensation due to past disputes over business administration at the enterprise (Fayyad, 2012a). In fact, many cases involved workers filing anonymous complaints in fear that employers would dismiss them from work.

Article 41 further provides some substantive and procedural conditions in order to practice such as provided below (Amarneh, 2008), and the failure to meet any of these conditions is deemed to be unfair dismissal, requiring workers’ compensation.

#### 3.1.1 Substantive conditions for enterprise restructuring

An enterprise must have reached an economic status obstructing its ability to shoulder the full burden of its obligations towards workers (Cowan, 2020; Dong et al., 2020; Eichenberger et al., 2020), as contractual relationships become cumbersome (Eaton and Heckscher, 2021; Rao et al., 2020). Hence, the Palestinian Court of Cassation held that technical reasons should be in place, requiring that an enterprise be restructured to ensure ongoing business activity. To this avail, some sections may be merged or closed. Some activities or business branches may be dispensed with, resulting in the abolition of certain posts and reduction of the number of workers in view of the pressure of expenses and performance enhancement.^6^ The court's role lies in validating how serious justifications are and monitoring how appropriate the reasons for terminating employment contracts are to those needs (Mansour, 2010). The judge has to observe employers' discretion, as to whether the justification they invoke requires that a worker be dismissed or another action can be in place to avoid their emergency conditions (Amarneh, 2008).

According to the Palestinian Court of Cassation ruling, loss must be incurred over several years for this right to be exercised: “The employer’ financial loss in a single year is not a justification for terminating the employment contract because business oscillates between profit and loss”. Hence, the continuity of the loss over several years in succession must be established because of the general economic situation or because of a downturn that has affected him so that the termination of the employment contract can be justified.”^7^ Loss is verified by the employer, who provides evidence that the enterprise has been economically affected by the accumulation of bank debt as well as ongoing and steady decrease in turnover. It must also be proven that the enterprise is incapable of financing its activities and covering business expenses (Fayyad, 2012a). The closure provided for by the Law involves full shutdown of the enterprise activity, rather than a halt of operations for a temporary period of time. Therefore, the Jerusalem Court of Appeal dismissed a worker's claim to pay unfair dismissal compensation because the employer had proven the complete close of business at his enterprise, entitling him with the right to terminate employment contracts with no fixed term only.^8^

The field research findings have also unveiled a divergence of views among legal experts regarding the meaning of substantial loss, which authorizes the employer to use Article 41 of the Labour Law. According to an academic, the provisions of Article 41 are vague and unclear, and this is the reason behind its different interpretations. Another academic reported that Aarticle 41 concerns indefinite employment contracts only, while other jurists reported that the concept of “substantial loss” entails the erosion of capital in a manner that puts at risk the existence and ability of an enterprise to maintain business operations in the market. A lawyer reported that he had a negative experience with the Ministry of Labour (MoL) in many labour cases regarding to Article 41; the MoL does not intervene in any decisions against employers even if it finds that they wrongfully terminated the employment contract, because sanctions provisions under the Labour Law do not make any reference whatsoever to Article 41. A trade unionist confirms that technical reasons apply to a substantial loss of capital, rather than profit while some jurists added that public disasters and international economic crises are not considered a reason for laying off workers from enterprises on grounds of restructuring (Amarneh, 2008).

#### 3.1.2 Procedural conditions for restructuring

Exercising this right requires the employer to notify the MoL about the enterprise restructuring. The Ramallah Court of Appeals interpreted this condition in a broad approach, holding that the employer is not exempted of notification even when an enterprise is closed down due to failure to fulfil its obligations either by an administrative decision or a decision made by its owner. Otherwise, the employer's decision on the termination of employment contracts is deemed to be unfair.^9^ The Court of Cassation rationalises this obligation on the grounds that the MoL should be capable of exercising its oversight role vis-à-vis the seriousness of these justifications. If the employer fails to inform the MoL, the court will have the discretionary power to assess how serious those justifications are with a view to barring arbitrary decision-making by employers.^10^ However, the field research findings have revealed a clear flaw in this interpretation; Jurists have significantly divergent views of this approach of the Palestinian judiciary. As one judge reported that the lack of a legislative provision that defines the MoL role in oversight of the validity of actions taken by employers makes the notification worthless, so failure to give such a notice is, therefore, devoid of any legal effect.

By contrast, an academic reported that the Palestinian judiciary does not embrace a unified approach in dealing with the legal effect arising from the lack of notification. While the Court of Cassation deemed that the decision on restructuring was null and void in the event a notification is lacking, it ruled otherwise for other similar cases providing that the purpose of notification is to inform the MoL only and does not influence the validity of the decision. Many academics reported the same confusion either because, they add, Article 41 is unclear and causes confusion over the purpose of the notice (i.e. notification or oversight). On the other hand, an academic is of the view that Article 41 should be interpreted in light of the purposes and goals of the law as a whole. If the Law is intended to protect workers' rights, the objective of the notice has to ensure that the MoL oversee employment conditions and be entitled to assess whether the employer's decision is valid or not. Agreeing with this academic view, some lawyers made clear that, in principle, the decision on dismissal is unfair in the event it does not comply with the substantive conditions provided for by Article 41. The purpose of the notice is to update the MoL with the fact that employer’ decision is not unfair (Fayyad, 2014), consequently giving rise to the Ministry's right to review how valid this decision is.

It has to be noted that the Law does not require that notification of the MoL precede the termination or suspension of employment contracts. This suggests that the legislature intends to bind employers to notify the MoL in light of the extraordinary circumstances that face their enterprises. It does not place as a condition that the notice antedate the termination or suspension of all or some indefinite employment contracts. It is also noted that, contrary to Arab legislation, the Palestinian Labour Law does not provide that the MoL has the oversight power to determine the lawfulness of such a decision. For example, Article 31(b) of the Jordanian Labour Law authorizes the Minister of Labour to establish a committee of the three production parties to validate the actions taken by employers within a period of 15 days from the date of notification. This ensures that oversight is exercised *a posteriori* to examine the validity of these actions.^11^ The Minister of Labour issues this decision within seven days from the date of receipt of the recommendation, which either approves of the employer's decision or requests that the employer reconsider the decision made against a worker. In case the employer does not abide by this notice, the power transitions to the court in order to prevent the employer from abusing this right.^12^ In addition, employers must notify workers with the decision within one month after it is made. Employer are required to pay to workers the allowance in lieu of notice if they do not implement this obligation.^13^

Based on the Palestinian Court of Cassation's approach, the legal effect arising from noncompliance with all these conditions does not involve rescinding the dismissal decision. Rather, dismissal is deemed to be wrongful, consequently establishing the workers' right to unfair dismissal compensation.^14^ “Whereas the defendant dismissed the plaintiff from employment without prior notice and did not notify the MoL of the economic conditions which necessitated the termination of the plaintiff's employment contract in order to validate the actions, thereupon, dismissal of the plaintiff is wrongful. The plaintiff has the right to claim the unfair dismissal compensation established by law”, the same Court ruled.^15^ Apart from this ruling, if dismissal is caused by trade union activity, the worker must be reinstated.^16^ Reference cannot be made to the general rules of contract law; termination cannot be justified by the fact that it is based on the dissolution of the contractual relationship by the force of law. Implementing the obligation is impossible in view of the enterprise shutdown. The conditions for dissolution imply that full implementation of an obligation is impossible (Abu al-Leil, 1998). This did not occur in the cases in question because business interruption at the enterprise was temporary (Fayyad, 2012a).

In practice, the field research has reported that many workers were deprived of compensation because substantive and procedural conditions were inadequately applied. In one case, a teacher was even denied unfair dismissal compensation, including the sum of salaries of 24 months (US$ 28,000), based on the provisions of Article 47 of the Labour Law.

### 3.2 Modifying employment contract to the detriment of workers’ interests

The field research has shown that employers took advantage of the COVID-19 pandemic to modify employment contracts as follow (see Table 1):

⁃ Workers were forced to take unpaid leaves (37%).
⁃ Workers were obliged to agree on reducing their wages or threatened with dismissal (19%).
⁃ The nature of employment was substantially changed, e.g., a restaurant employee at a hotel was forced to work at a subsidiary company to maintain and paint sidewalks in a remote city or on agricultural fields in another city (12%).
⁃ Wage calculation was modified, against workers' interests, to an hourly, piece, or per diem basis instead of monthly employment (18%).

Of note, most of these actions are a clear contravention of the Palestinian government decisions and tripartite agreement mentioned below. This was explicitly confirmed by an employer: “We have been committed to the tripartite agreement. After it had expired, we laid off workers and gave them the option of an unpaid leave or a severance pay. We paid their compensations without unfair dismissal benefits. We sufficed with the severance pay.”

It is noted that the unlawfulness of the first and second measures do not give rise to any judicial or jurisprudential disputes. Both actions clearly run counter to the provisions of the tripartite agreement, presented in detail below. Therefore, this section only outlines the nature and impact of the third and fourth actions.

#### 3.2.1 Substantial changes for the nature of employment

According to field research findings, employers have markedly modified the place and nature of employment agreed to under the employment contract. Workers were threatened with dismissal if they did not comply with these decisions. Many cases were monitored, where monthly wage workers were forced to shift to per diem or hourly employment. This forced many workers to change the nature of their work. For example, a hotel employee in the city of Bethlehem was forced to work at a subsidiary of the holding company in the Jericho Agro-Industrial Park to repair and paint sidewalks. A month later, that worker was relocated to some restaurants in the Rawabi city, Ramallah. In the Bethlehem city, a restaurant owner forced employees to work at palm plantations and pick dates in Jericho. Employees agreed to do so under the threat of dismissal.

Of particular note, Article 32 of the Labour Law prohibits that a worker be assigned to a work, the nature of which substantially differs from that agreed to in the employment contract, unless necessity requires performing such work, on condition that it is temporary. The worker may leave employment following a notice to the employer, while retaining their legal rights, including severance pay and rights generating from employment in a work, the nature or grade of which markedly differ from those agreed to under the employment contract. A review of court decisions shows that this prohibition applies to cases, where the nature of the work assigned varies from the agreed employment under the contract. If there is no difference, the worker may not invoke this prohibition. To this avail, in 2017, the Ramallah Court of Appeals held that a school's decision, which required that the plaintiff to teach students in contrariety with the agreement under the employment contract (namely, to hold the position of a director and supervisor of the school's kindergarten), did not contradict the provisions of Article 32 of the Labour Law. Teaching at the school was an integral part of the enterprise business. No substantial change was introduced to the nature of work, particularly given that the wage was not altered.^17^ Accordingly, many cases reviewed by the field research team explicitly indicated a substantial difference in the nature of assigned work. While a contract provided for work in a restaurant, workers were employed in the agriculture sector. Others were employed in street construction work despite the fact that their contracts provided for employment in the hotel industry. These cases were clearly in violation of the Labour Law. As a result of these decisions, affected workers must be entitled to unfair dismissal compensation. As judgement to this effect is not contingent on a particular period of service at an enterprise. Workers are entitled to compensation even if the period of their service is less than a year.^18^

#### 3.2.2 Modifying method of wage calculation

Field research findings showed that, in many cases, employers modified wage calculation rules against the interest of workers through the following means:

⁃ Changing the wage calculation method from work on a monthly or per diem to hourly basis.
⁃ Modifying the wages of workers, particularly high earners, on the pretext that employers were unable to pay due to declining income of enterprises. This took place by threatening workers with dismissal if they would not agree to the reduction or by dismissing the worker and terminating his contract and later sending someone to convince him services for the purpose of pressuring him later and sending someone to convince him to return to work for a lower wage. “Later, the employer offered that I work for lower pay, but I refused”, a teacher reported.

The Court of Cassation prohibited modification of the method of calculating the agreed wage individually “If the worker agrees in writing to be transferred from monthly employment corps to the status of those who earn wages differently, such a modified wage calculation method is contrary to the law and must not be applicable. In such case, the worker shall maintain the right he has gained throughout the period of his employment on a monthly wage basis.”^19^

Even in cases where it is in the interest of workers, a modification is unlawful as long as it is not explicitly approved by the worker. Wage is determined by the agreement of both parties. It may only be modified by their mutual agreement even if it confers on the worker some privileges provided for by the Labour Law, such as enjoyment of the days of rest on which workers are off during the month.^20^ Based on this protection, the Kuwaiti Amended Labour Law of 2010 deems that any agreement aimed at modifying the wage to the disadvantage of workers and reached prior or subsequent to the contract's entry into force is totally null and void as a matter of public policy (Al-Uteibi, 2013).^21^ The legislature further prevents transferring a monthly wage worker to another category without their written consent, on condition that the worker is not financially affected by such a modification.^22^ However, this provision is not applicable in Palestine.

Moreover, according to Article 7 of the Arab Convention No. 15 of 1983 concerning the determination of and protection of wages, approved by the Arab Labour Conference in March 1983, “[t]he worker is entitled to full wage remuneration even if he did not perform work for reasons beyond his control, provided that national legislation states those reasons.” Also, Article 37 of the Arab Convention No. 6 of 1976 concerning Labour standards (revised) provides that “[t]he employer may only make a deduction from the worker wages in accordance with the conditions and limits, which must be provided for by the legislation of each state.” Article 38 further states that “[w]orker wages are a privileged debt in their entirety and is owed by the employer. It has priority over all other privileged debts.” Therefore, the Ramallah Court of Appeal considered that the employer's conduct (giving workers the option to reduce their wages or terminate their employment contracts on grounds of the economic loss affecting the enterprise) was unfair dismissal even if the employer notified the MoL thereof. In this context, the Egyptian Court of Cassation considers that the employer's offer to the worker to return to his work with a lower wage after the termination of his work contract indicates that he was exerting pressure on the worker to force him to reduce the wage, and therefore the decision to terminate the contract is considered unfair termination.^23^

## 4 Assessment of trade union and MoL's response to the pandemic impact

According to field research findings, workers and employers were not satisfied with the MoL and trade unions' response to the COVID-19 pandemic impact. Dissatisfaction was also voiced at the decisions taken by employers against workers. Paradoxically, dissatisfaction was expressed by many trade union representatives, who were unhappy with the MoL measures; it was further reflected in these representatives' personal assessment of handling workers' complaints and requests. Everyone was not satisfied with the performance of Palestinian courts, negatively affecting workers' decision to have recourse to regular courts and institute cases against employers. The field research has noted that all interviewees were unanimous in pursuing amicable solutions in the settlement of disputes arising from employer decisions because of slow formal judicial proceedings. Some workers wished to maintain amicable relations with employers in the hope of job reinstatement. A worker reported that he did not object to the payment he received because of his dire need for money. A trade union representative added that, in view of inefficient Palestinian judicial process and lacking alternative employment opportunities, workers were forced to submit to employer decisions. According to another representative, recourse to courts is interminable; “the fact that specialised labour courts are lacking prompts us to seek amicable solutions, albeit to the detriment of the worker’ rights.” This representative also indicated that his trade union branch office and Labour Office in the city of Hebron had received 1,200 complaints. A considerable number of these complaints were resolved amicably with employers. By contrast, a worker reported that he retained a lawyer to prosecute his employer, and when he was asked about the outcome of the case, he reported that he had no idea.

### 4.1 Assessment of trade unions’ performance

The field research findings have demonstrated that trade unions played an inadequate role in protecting workers’ rights vis-à-vis employer decisions. Curiously, interviewed trade union representatives were also in general agreement on this assessment. Reported deficiencies were featured in several aspects.

Firstly, interviewed workers were not aware of the existence of trade unions because of the failure of these unions to effectively make their presence visible. According to a worker, “I am not aware of any trade unions, which I can resort to. I did not think about it.” Another said: “In fact, I have no idea that I belong a to particular union. I was not aware of any unions for private schools' employees. Hence, I did not have recourse to any agency. No government body or trade union communicated with me regarding my case. So far, I have not resorted to anybody. A person from the labour movement called and told me that he would adopt my case, but nothing has happened.” A representative of employers further stated that there was no coordination whatsoever between workers and trade unions. He asserted that all workers at his enterprise did not know that trade unions existed. For them, trade unions were mere designations that did not exist on the ground. Table 2 below shows that 130 out of 297 (43.8 %) workers were strongly dissatisfied with trade unions’ performance, 87 (29.3 %) reported that they were not satisfied (see Table 2).

**Table 2:** Assessment of trade unions’ performance.

**The Employee belongs to a trade union (297 Respondents)**	
Yes	118 (40%)
No	167 (60%)
**A trade union communicated the Employee (297 Respondents)**	
Yes	48 (16%)
No	246 (84%)
**The Employee knows how to communicate the union (297 Respondents)**	
Yes	117 (40%)
No	177 (60%)
**The Employee is satisfied with the performance of the union (297 Respondents)**	
Strongly agree	6 (2%)
Agree	25 (8.4%)
Disagree	87 (29.3%)
Strongly disagree	130 (43.8%)
I do not know	49 (16.5%)

Secondly, the field research noted that many employers were totally unresponsive to trade unions' attempts to reach an amicable settlement to labour disputes. A dismissed worker reported that the Secretary General of the Palestinian General Federation of Trade Unions (PGFTU), communicated with his employer (a wealthy and influential businessman) and made an extensive effort to resolve the case. However, the employer was unwilling to engage in any dialogue. In another case, a worker said the refugee camp committee intervened and arranged for a meeting with the employer. Nevertheless, the latter excused himself from making it to the meeting and refused intervention. In many other cases, trade unions explained they could not exert pressure on employers to redeem workers’ rights. They only advised workers to have recourse to court.

Thirdly, many trade unionists reported that no unions took the initiative to provide free-of-charge hotlines to workers or even build databases to document decisions on the unfair dismissal of workers. As a consequence, many workers’ rights have been violated. This view is in agreement with field research statistics: 177 out of 297 (60 %) workers reported that they did not know how to communicate with trade unions in order to lodge complaints.

Fourthly, coordination was not in place between trade unions and relevant government bodies. According to a trade union representative, “[i]n some cases, coordination was only limited to personal relationships between the representatives of both parties.” Another union representative said: “Oftentimes, government bodies deliberately excluded the PGFTU from any official coordination for partisan reasons. As another representative put it: “The lack of coordination emerged in the way aid was distributed to the families of affected workers by the MoL. Aid was distributed randomly without relying on databases that equitably identify the needy groups.”

According to a trade union representative, the reason behind the weak performance of trade unions could be attributed to the absence of a law that regulate trade unions in Palestine. Union activities are generally dominated by partisanship and political quota system. Another unionist asserted that insufficient financial resources negatively impacted union functions. The free market system of the Palestinian Authority (PA) has placed increasing financial burdens on poor households, particularly given that unemployment has risen to 14%, and could jump up to 30 % after the COVID-19 pandemic.

Findings of the field research are in line with many studies released by specialised research centres over the past two decades. Providing an overview of inadequate union activity in Palestine (Table 3), these publications account for weak union performance regarding workers’ rights representation (Ladadweh, 2019). This situation has created a gap between unionised workers (those who pay financial obligations to unions and have the right to stand as candidates for membership and election) and unregistered workers, as follows (The Numbers of Workers Who Belong to Trade Unions in Palestine, 2020):

**Table 3:** Number of unionised workers.

Trade union	Registered workers	Unionised workers
PGFTU	287.000	170.000
Palestine Workers Union	150.000	45.000
Federation of Vocational Unions	20.000	10.000

The reason of this deficiency can be mainly attributed to a legal vacuum in governing unions activities in Palestine. According to the Director General of the MoL Labour Relations Department, the MoL finds itself dealing with over 300 conflicting trade unions (The Legal Regulation of Union Activity in Palestine, 2011). Additionally, the fact that political parties are in control of, and interfere with, union activity has turned trade unions into more a representative of those parties than of the interests of unionised workers. This featured prominently after the internal political divide, which affects the Palestinian territory (Abdul Majid, 2017). Political conditions have also played a negative role in union activity across the occupied Palestinian territory. The trade union movement continues to be crippled by Israeli harassment and prosecution. Also created by the Israeli occupation, the current economic downturn has been caused by economic blockade, land grab, and confiscation of property, further increasing unemployment rates and prevalence of poverty. According to 2017 statistics of the Palestinian Central Bureau of Statistics (PCBS), some 13% of Palestinian workers were employed in the Palestinian territory occupied in 1948 (Abu Amer, 2018).

### 4.2 Assessment of the MoL performance

According to field research findings, interviewees agreed that the MoL management of the crisis was unsatisfactory. This involves the handling of workers’ complaints, coordination with trade unions, and pressure on employers to reverse wrongful decisions against workers. In this context, a worker stated: “I filed a complaint at the Labour Office, but no one has contacted me ever since.” Other workers reported that employers refused any bargaining and rejected to comply with the MoL decisions. A worker said that the MoL did not pay attention to workers or strike a balance between the interests of workers and employers. Table 4 below monitors dissatisfaction among workers and employers.

**Table 4:** Assessment of the MoL performance.

**The Employee is satisfied with the MoL (297 Respondents)**	
Strongly agree	6 (2%)
Agree	20 (6.7%)
Disagree	139 (46.8%)
Strongly disagree	44 (14.8%)
Do not know	
**The Employer's evaluation for the MoL's cooperation (78 Respondents)**	
Satisfied	2 (2.6%)
Not satisfied	57 (73.1%)
Do not know	19 (24.4%)

Commending the MoL efforts, the MoL Undersecretary said that the Ministry set free-of-charge hotlines to receive complaints and document data on workers. The MoL also developed databases, which included many workers. The MoL Undersecretary highlighted coordination with trade unions (L.-P. Beland et al., 2020, Beland et al., 2020; Lemieux et al., 2020). However, this claim is incompatible with statements made by employers and trade union representatives, who agreed that lines of communication with the MoL were off during the lockdown period. Further, the MoL decision-making process was in disarray. In addition to lacking official coordination between various institutions, response to the COVID-19 pandemic was clearly inadequate and poorly managed. According to a representative of an employers' organisation, the Ministry's role was inadequate, and it did not fulfil its duty properly.

Perhaps the most important achievement announced by the Ministry was the so-called Tripartite Agreement, which involved the MoL, Palestinian General Federation of Trade Unions (PGFTU), and a representative of the Private Sector Coordinating Council (PSCC). Concluded on 16 March 2020, the agreement provided that private employers, whose enterprise business came to a complete halt after the state of emergency had been declared, would pay 50 % (no less than NIS 1,000) of the March and April salaries. Remaining wages should be paid after the crisis would come to an end. According to the agreement, employers who were not affected by the pandemic should pay full wages to respective workers.

The MoL considered this agreement a great achievement in dealing with the crisis. Although it was not fully satisfied, according to the MoL Undersecretary, the Ministry announced that the agreement represented the maximum which could be paid by employers affected by the emergency declaration and business shutdown. Reporting to the *Palestine Today* News Agency, the Minister of Labour emphasised that the agreement guaranteed the rights of both workers and employers. This was further confirmed by the PSCC representative. The PGFTU Chairman elaborated that “[t]his agreement serves thousands of male and female workers, enabling them to attend to the necessities of their and their families’ daily lives” (A Tripartite Agreement to Regulate the Work of the Private Sector during the Corona Crisis, 2020). However, few academics agree with this assessment, believing that the agreement was a great success scored by the Ministry. While protecting employment contracts against termination due to the pandemic, the agreement largely represented interests of private sector employees.

Field research findings have demonstrated deep divisions over the legality of, and employers’ compliance with, the tripartite agreement. Many questions were raised, viewing the agreement as a permission for employers to break the law and terminate employment contracts.

#### 4.2.1 Challenges of the agreement’ implementation

According to field research findings, these challenges can be summed up as follows:

##### 4.2.1.1 Stakeholder representation’ challenges

Field research has found out that parties to the tripartite agreement did not fully represent all workers and employers in Palestine; this was evident in a large number of press releases from many stakeholders, who rejected the agreement. According to a representative of employers, “[n]o one asked us. Like the public, we just heard about the agreement. They did not communicate with us.” Justifying the agreement, the MoL representative said it was customary that the Ministry would approach the PGFTU and Federation of Palestinian Chambers of Commerce, Industry and Agriculture as representatives of both parties. However, Similar to the findings of (Bader, 2018), the official statistics showed that many other trade unions were not represented in the agreement. Lawyers, academics, and judges are in consensus that the tripartite agreement is only binding for workers affiliated with the PGFTU, a party to the agreement. These workers constitute a significant proportion of private sector employees. According to an academic, the Labour Law (Article 54) includes provisions that regulate collective bargaining with a view to improving working conditions and increasing production efficiency. As long as it does not concern these matters, the agreement is not legally binding. However, some academics disagree with this perception, arguing that an agreement on worker wages and protection of employment contracts contributes to enhancing working conditions. In essence, the agreement is legitimate despite the fact that it is available only to those represented by the PGFTU. Table 5 below shows that, compared to 62% of the workers, 82% of the employers were not aware of the existence and content of the agreement.

**Table 5:** Assessment of the tripartite agreement.

**I know the existence of the tripartite agreement**		
Employees (297 Responded)	Yes: 111 (38%)	No: 182 (62%)
Employers (78 Responded)	14 (18%)	No: 64 (82%)
**Fairness of tripartite agreement**		
Employees (111 Responded)	Fair: 3 (2.7%)	Unfair: 107 (97.3%)
Employers (14 Responded)	Fair: 4 (28.5%)	Unfair: 8 (57%)
**The enterprise implemented the tripartite agreement**		
Employees (111 Responded)	Yes: 6 (5.4%)	No: 100 (90%)
Employers (14 Responded)	Yes: 5 (35.7%)	No: 9 (64.3%)
**The employer paid the remainder of the worker's dues according to the tripartite agreement**		
Employees (111 Responded)	Yes: 6 (5.4%)	105 (94.6%)
Employers (14 Responded)	Yes: 4 (28.5%)	No: 10 (71.5%)

##### 4.2.1.2 Fairness of the agreement

Representatives of workers, employers, trade unions, and some political parties other than the governing party generally agree that the tripartite agreement was not fair (Shahin, 2020). On the other end, an academic was of the view that the agreement represented workers' interests because it maintained the contractual relationship with employers (Fayyad, 2019). It did not deprive workers of any portion of their earnings, but postponed payment of half of the wages until the pandemic is over. Table 5 above reflects workers and employers’ major dissatisfaction with the fairness of this agreement.

As noted by field research workers considered on many occasions that the agreement permitted employers to terminate employment contracts after the agreed the two-month period expired. According to an employer, “[a]fter the tripartite agreement had expired, we suspended workers and gave them the options to either go on unpaid leave or receive severance pay. We paid workers’ compensations. We did not pay unfair dismissal compensation and sufficed with the severance pay.” The reason could be attributed to vague content and implications of the agreement. In the first place, it did not set a clear classification standard for affected enterprises, consequently providing a cover for employers to interpret the agreement as they wished under government patronage. Following the three-month period, the MoL proposed a new agreement, but it was turned down by private sector representatives.

##### 4.2.1.3 Economic challenges

the agreement caused financial damage to workers; economic statistics showed that noncompliance with the agreement could constitute a financial catastrophe for workers. According to the Palestinian Central Bureau of Statistics (PCBS) 2017 survey results, workers’ average monthly income was NIS 3,000, so the enforcement of the agreement would bind employers to pay an average monthly salary of NIS 1,500, which is less than the extreme poverty line (NIS 1,974) (Sileus, 2020). The agreement also set the minimum payment at NIS 1,000 per month, i.e. half of the poverty line amount. Statistics released by relevant bodies in Palestine showed that small enterprises (SEs) employing one to four workers comprised 88.6% of the private sector. As clearly demonstrated by field research findings, SEs were incapable of paying wages of some 260,000 workers during the period of suspension.

Obviously, these figures are compatible with field research findings. Table 1 above indicates that 42% of affected enterprises employed less five workers, while others with less than one worker represented 22%. Table 1 above shows that 206 out of 297 (70%) affected workers earn less than NIS 4,000 per month. Thus, half of the salary to be paid to this large section would not be sufficient to cover their personal and household needs at all.

##### 4.2.1.4 Employer compliance

The field research has revealed that a small portion of enterprises complied with the tripartite agreement. According to a worker, “[t]he company adhered to the agreement; it gave us full wages for the two months as provided for by the tripartite agreement. At the same time, however, it deducted and counted our annual leaves as part of the suspension period.” An employer reported that “[i]n spite of our significant loss, we committed to the tripartite agreement during its validation period only.” A trade union representative reported: “We did not evidently notice implementation of the agreement on the ground; an abusive conduct by employers, who dismissed a number of workers claiming that they suffered hefty losses, was reported in many cases.” A worker explained that the employer neither complied with the agreement nor paid half the salary. Additionally, some employers took advantage of the agreement and applied it to their workers even though their enterprises were not affected by the pandemic. The authors agree with a trade union representative, who reported that problematic enforcement was visible at small enterprises (representing 92% of the private sector) (“The Terms of the Agreement between the Ministry of Labor,” 2020) as employers were unable to cover workers' wages over the suspension period. Table 5 above demonstrates that 94.6 % of employers, who enterprises were affected, did not abide by implementing the tripartite agreement either because they were not aware of or dissatisfied with it. Also, 71.5 % of employers reported that they were not committed to paying workers' benefits as provided by the agreement. These figures match workers’ statements. 100 out of 111 workers (90%) reported that employers did not apply the agreement, and 105 out of the 111 workers (95%) did not receive the rest of their benefits.

#### 4.2.2 The assessment of the agreement’ legality

As noted by the researchers, legality of the agreement was a major point of contention between members of the legal community (judges, lawyers, and academics) and civil society representatives.

##### 4.2.2.1 Opponents to the legality of the agreement

Some argue that the tripartite agreement was both unlawful and illegal in its entirety. According to the Palestinian NGOs Network (PNGO), the agreement contravenes Article 6 of the Labour Law, which does not permit waiving any approved workers’ rights; no party may breach or abandon these rights (A Human Rights Activist Criticizes the Tripartite Agreement on Workers in the Private Sector, 2020). Some trade union representatives were of the opinion that the agreement was explicitly in violation of Article 38 of the Labour Law, which provides that an employment contract shall not be terminated in the event of the issuance of an administrative or judicial decision closing the enterprise or temporarily halting its activity for a period of time not exceeding two months. This was expressly highlighted by the Jerusalem Court of Appeals in Case No. 81/2016.

Pursuant to Articles 110–114 of the Basic Law, declaration of the state of emergency does not legally authorise the MoL or government to repeal, amend, or suspend valid legal provisions (Sileus, 2020). In such a case, the legislature also obliges employers to fulfil their obligations towards workers if the period of lockdown does not exceed two months (Sultan, 2012). Deviating from economic character of wages, the legislature comes closer to the social perception, which refuses that worker stay without pay even at the expense of employers (Al-Hindyani, Khaled, 2010). A trade union representative summed up the agreement as reflecting “a political reality, originating in power and creation of a *fait accompli*. It runs counter to all the requirements of justice sought by law.” Judges, lawyers, and academics agreed that such an association was invalid. Article 38 concerns the closure of enterprises by an individual administrative or court decision as a punitive action or precautionary measure (Coronavirus Declared a Pandemic as Fears of Economic Crisis Mount Financial Times, n.d.). A general lockdown decided by the MoL or Council of Ministers in response to the pandemic consequences does not apply to enterprises.

Some academics and judges agree with this approach, providing that a worker's waiver of some or all their current and future rights is null and void; it does not bar workers' right to claim these rights at a later stage. This is driven by the nullity of any condition under a contract or agreement, according to which a worker abandons any right granted by the Labour Law.^24^ This is to protect the worker from any employer’ pressures for the purposes of waiving his rights established by law.^25^

##### 4.2.2.2 Proponents of legality of the agreement

Contrariwise, some interviewers argue that the tripartite agreement is lawful because it represents workers' interests, justifying their approach on the fact that the COVID-19 pandemic can be characterised as “Unforeseen circumstance” which entitle employers to suspend their contractual obligations towards workers during the lockdown period. “What's wrong if we collect half of workers' salaries at a time when employers insist not to pay wages in full,” the MoL Undersecretary commented. Another academic elaborates that: comparative law jurisprudence recognises the theory of “temporary suspension” of the contract to maintain balanced contractual relationships, preventing revocation which may result from a party's inability to perform his/her contractual obligations, without influencing the existence of the agreement (Al-Ayyash, Murdhi, and al-Huthal, 2020). Distinctively, the notion of temporary suspension achieves collective interest in stable labour relations despite temporary business interruption. It, therefore, requires the following conditions (Al-Ayyash, 2014): the bar to execution of the employment contract is temporary; the reason behind failure of execution is alien; the period of execution is not a fundamental element of the employment contract; and the contract does not include a resolutory condition. If these conditions are met, the employment contract is suspended, and so is the enforcement of obligations, including wage payment, until such time the cause of suspension ceases to exist. These do not affect the continuity of the contractual relationship between both parties.

Some legal experts align with this approach; they argue that the temporary suspension of contracts is in tandem with the theory of *Al-‘Udhr* (excuse), adopted by the Hanafi School of Islamic jurisprudence. Reflecting a direct application of the theory of “Unforeseen circumstance”, which the Hanafi jurists explicitly apply to many contracts, such as leases and supply contracts. “An excuse” is any incident that causes damage to the contractor in himself or his property as a result of the contract, whereby the subject matter of the contract can only be fulfilled by a damage incurred by the contracting party in himself or in his property. It is also in consistence with the general principles outlined by the *Mejelle* (Ottoman Civil Code), namely, Articles 19 (Injury may not be met by injury), 27 (Severe injury is removed by lesser injury), 30 (Repelling an evil is preferable to securing a benefit), and 31 (Injury is removed as far as possible (Mansour, 1993).

Calling it the theory of *Al-Jawa'ih*, the Maliki School of Islamic jurisprudence applies this principle as well. According to *Al-Qawanin al-Fiqhiyyah* [Jurisprudential Rules] of Ibn Juzayy, “If someone purchases a crop, but it is afflicted by an intervening contingency, he shall be relieved of as much price as the quantity affected by the intervening contingency. The price is deducted under two conditions. Firstly, the intervening contingency is not manmade, such as drought, excessive rain, cold, wind, locust, and so forth (Al-Sanhouri, 1998). The army and thief are a point of contention. Secondly, the intervening contingency affects one third of the crop or more (Shalbik, 2007).”

Both theories (*Al-‘Udhr* and *Al-Jawa'ih*) are not codified under the *Mejelle* obviously because *Sharia* [Islamic law] jurists were probably not interested in legislating general theories. Rather, they addressed each case separately and exercised discretionary power to reach the legal rule in consistence with the requirements of the *Sharia* and justice (Qabbani, 1980), deriving from a special provision or exercising discretion on the grounds of legislative rules or reasonable texts. They apply themselves assiduously to analysing incidents both scientifically and factually (Fayyad, 2013), taking into consideration the surrounding circumstances in any age, which they believe has to do with constituting the effective cause of the rule (Al-Sanhouri, 1959). Against this background. As-Sanhouri called for explicitly including the theory of intervening contingencies in our Arab legislation in reference of the theory of excuse, derived from the Hanafi School. According to As-Sanhouri, “[t]his theory is fair. In their new legislation, the Egyptian legislature can apply it on the basis of the theory of necessity in *Sharia*. It is a theory with a broad scope (Al-Sanhouri, 1959).”

The suspension effected by a decision-making process is temporary and vanishes when the relevant reason ceases to exist (At-Tourah, 2020). By definition, suspension does not affect existence of the agreement; contractual obligations are not executed during the accident period. In other words, it reflects a temporary stay of obligations (Al-Hindyani, 2000), so while the worker is not committed to work, the employer is not bound to pay wages during the period of suspension. It is an outcome of the general rule: “pay for work”.^26^

Of note, modern comparative jurisprudence is in line with the approaches of many Islamic jurisprudence scholars (Fayyad, 2012b). For example, in relation to the changing value of money, prominent Islamic scholar *Ibn Abidin* stresses the necessity of reconciliation between the contracting parties in order to share the contingent burden among them. To this avail, the seller and buyer, or creditor and debtor, will share the damage arising from the changing value of money. According to Ibn Abidin: “[i]f what is valued at one hundred becomes equivalent to ninety of a kind, ninety five of another kind, and ninety eight of another, if we oblige the seller to take what is equivalent to ninety of the one hundred, damage will be limited to him. If we force the buyer to pay ninety for it, damage will be exclusive to him. Therefore, a compromise will be reached on the average (Abidin, 1875).” *Al-Hattab* elaborates: “*Ibn Abu Zayd* was asked if it rains, preventing a construction worker from working during part of the day. He said that he shall be paid for what passed and the rest of the day shall be revoked (Al-Maghribi, 1992).” Ibn Taymiyyah said: “If a premise is leased and the lease of which is of public use, such as a bath, hotel, and so forth, and the known benefit diminishes, such that neighbours of the premise move, customers decrease due to a fear or destruction, or a person in authority transfers them, the tenant shall be relieved of the rent to the extent of diminishing known benefit (Taymiyyah, 2004).”

In light of these jurisprudential approaches, in 1398 Hijri Calendar (1978 (, Session 1, 1398 AH, p. 99–104, the Islamic Fiqh Academy, decided as follows: “In contracts with lax execution (such as procurements, contracting works, and leases), if the conditions, other than circumstances, costs and prices, under which the contract was concluded change considerably due to public emergency causes which were not anticipated at the time of contracting, whereby execution of the contractual obligation incurs on the obligor unusually grave losses due to the fluctuation of prices on trade routes, and that was not an outcome of omission or negligence on part of the obligor in the course of executing his obligation, the judge shall have the right, in this case, upon dispute and on request, to modify the contractual rights and obligations in a manner that distributes the excessive amount of the loss caused to the contractor to both contracting parties.”

##### 4.2.2.3 Authors’ opinion

Authors disagree with the second approach and agree on the illegality of the tripartite agreement; the second opinion have turned a blind eye to the nature and scope of the excessive loss required by the theory of excuse which authorizes the aggrieved party the right to invoke it. The Legal Advisor to the West Bank-based Businessmen's Association, who was a member of the negotiating team, reported that they intentionally used the phrase *damaged enterprises* in the plural form to protect employers' interests and give them the right to take any actions, no matter how much damage was caused to them. Indeed, many employers based their decisions on this clause.

About the theory of excuse, the criterion of excessive loss is substantive; some Maliki School jurists set a clear arithmetic standard: the excessive loss caused by the pandemic must exceed one third of the economic enterprise capital (Rushd, n.d.). Others are of the view that it suffices that the pandemic makes repayment so onerous for the debtor that threatens him with an extreme loss; the cost would amount to encumbrance, whereby it will be unjust to force the contracting party to fulfil his obligations in full (Murqus, 1956; Sultan, 2012).

The one third refers to a third of economic enterprise capital, not just an opportunity for profit, which is missed as a result of reduced employment at the economic enterprise. Assessment of the encumbrance pertains to the subject matter of the contract, rather than to the contracting party itself. Accordingly, the Egyptian Court of Cassation ruled: “In conformity with Article 147 (2) of the Civil Law, a judge's intervention to revert the encumbrance posing a threat of the excessive loss is an authorisation by law; certain conditions must be met, most importantly, encumbrance that risks a heavy loss. Assessment of the risk is contingent on the substantive considerations of the deal itself (Boulihyah, 1983).” In another ruling, the same Court upheld: “Assessment of the cumbrousness affecting the debtor as a result of the pandemic falls within the ambit of the discretionary power of the court of merits. Such cumbrousness revolves around the substantive considerations of the deal itself, not the circumstances of the debtor (Kirah, 1964).” Assessment must be based on a consideration of an ordinary or average debtor; if the intervening contingency makes the obligation cumbrousness for the ordinary debtor, threatening him with an excessive loss (Fayyad and Byttebier, 2021), it is considered as such for the debtor, on whose part execution is required even though the loss is negligible in relation to his huge wealth (Qabbani, 1980).

Finally, many judgements stipulated that the loss lasts for a long and successive a period of time as a result of a public economic situation or downturn within the state. Accordingly, an employer's loss over several months does not entitle them to apply this theory.^27^ Field research statistics have shown that employers have illegally exploited the tripartite agreement. Setting the scope of loss was not subject to any oversight. Even in the worst affected sectors (hotels and restaurants), employers reported that loss involved a significant decline in profits. Enterprise capital was never affected. As the lockdown period did not exceed two months in total, it is inconceivable that economic enterprise capital was so affected that it would threaten the existence of enterprises. Table 1 above demonstrates that 44% of employers who took actions against workers indicated that their losses were medium, while 14% had minor losses 38% said their losses were grave.

## 5 Conclusion

This research has shown that workers have been largely affected by the economic damage caused by the COVID-19 pandemic in the West Bank; some were forced to take unpaid leaves (37%), others had pay cut or were threatened with dismissal (19%). Furthermore, the nature of employment was changed workers were compelled to work in another city (12%) and wage calculation was modified against the interests of workers (18%).

The study has concluded that workers who were employed by enterprises, which have never been affected by the pandemic (education sector and civil society organisations) were not immune from wrongful decisions, which claimed that enterprise capital was affected by the pandemic. The research has concluded that decisions made against workers were unlawful. The MoL and trade unions poorly addressed unfair decisions against private sector employees.

Many employers have taken advantage of the vague and poorly drafted Article 41 of the Labour Law, which allows employers to dismiss workers due to a loss that requires a reduction of the number of workers. When they applied this article, employers did not adhere to procedural conditions. According to the study, the enterprises did not suffer a great capital loss. It mostly involved a decline in the margin of profits earned by these enterprises in normal circumstances (62% of enterprises indicated that their losses were medium. Those who reported great loss (38 %) referred to the loss of profit, not of enterprise capital).

The research has also shown that many employers took advantage of the pandemic to dispense with workers’ services for retaliatory purposes. Others substantially changed the nature of employment agreed to under contracts or modified wage calculation rules against the interest of workers, expressly violating the law, judicial practice, and Arab Convention No. 15 of 1983 concerning the determination of and protection of wages, ratified by the Palestinian Authority.

The research has also evaluated the MoL and trade unions' performance. Results were unsatisfactory at all. While 44% of workers were considerably dissatisfied with trade unions’ activity, 29% were not satisfied with their performance. 60% of the workers reported that they were not aware how to get in touch with trade unions to report their complaints. The study has concluded that this deficiency is caused by the lack of legislation, which should regulate functions of the large number of trade unions. These unions are formed and operate on the basis of partisan, rather than professional, considerations. The executive also plays a major role in, and controls, the operation of trade unions.

Finally, the MoL has been widely criticised by all stakeholders (workers, employers, civil society representatives, and unionists) for the way it deals with the damage caused to affected workers. The Ministry has not established communication channels with, or built databases to monitor, affected workers. No decisions have been made to protect the rights of workers affected by employer decisions. Contrary to the MoL claim that it made a significant achievement in favour of workers, according to the study, the tripartite agreement did not reflect an adequate legal representation of all employers and workers. 62% of workers and 82% of employers were not aware of the existence and content of the agreement. By contrast, 82% of workers and employer respectively were aware of the agreement but expressed total disappointment and dissatisfaction with it. Moreover, many employers exploited the agreement to terminate employment contrast after it had expired. Field research has also concluded that the agreement used obscure and overbroad terms (e.g. affected enterprises), but did not determine the scope of damage, entitling employers to take advantage of the agreement against the interests of workers. This runs counter to the condition of grave loss to the enterprise capital, required by the theory of excuse or intervening contingencies in Islamic law as a parallel application of the theory of emergency conditions adopted by contemporary laws.

This research recommends the lawmaker to: amend Article 41 of the Palestinian Labour Law, providing explicitly for the unlawful use by employers of their right to reduce employment at enterprises without obtaining prior approval from the MoL; provide clearly for considering a decision on the dismissal of some workers for the purpose of reducing employment to be unfair if the employer does not meet procedural and substantive conditions under the law; initiate cooperation between the MoL and Ministry of National Economy to develop databases to identity those enterprises affected by the pandemic and determine the percentage of damage; provide MoL free-of-charge hotlines to workers and build databases to identify workers affected by employer decisions; ensure that all workers and employers are represented in any future agreements on the rights of private sector employees; regulate trade unions' performance through special legislation, maintaining their independence remotely from partisan pressure and executive interference; establish specialised labour courts to provide summary disposition of labour disputes; avoid using overbroad terms in any future agreement on workers’ rights and determine grave loss of enterprise capital as a substantive control, which justifies enterprise decisions affecting relevant workers.

## Declarations

### Author contribution statement

Pieter Sahertian: Conceived and designed the experiments; Analyzed and interpreted the data.

Umiati Jawas: Performed the experiments; Contributed reagents, materials, analysis tools or data; Wrote the paper.

### Funding statement

This research did not receive any specific grant from funding agencies in the public, commercial, or not-for-profit sectors.

### Data availability statement

Data included in article/supplementary material/referenced in article.

### Declaration of interests statement

The authors declare no conflict of interest.

### Additional information

No additional information is available for this paper.

## Footnotes

1 Court of Cassation, Civil Judgements, Objection No. 1545 of 1998, p. 2206.

2 Judgement No. 39/2019, Jerusalem Court of Appeals, 29 April 2019.

3 Judgement No. 30/2016, Jerusalem Court of Appeals, 30 May 2016.

4 Judgement No. 1250/2018, Jerusalem Court of Appeals, 25 March 2019.

5 Judgement No. 656/2018, Court of Cassation convening in Ramallah, 1 July 2019.

6 Decision No. 92 of 2008, Palestinian Court of Cassation, (Five-judge panel), 29 June 2009.

7 Decision No. 92 of 2008, Palestinian Court of Cassation, (Five-judge panel), 29 June 2008, available at AL-MUQTAFI: http://muqtafi.birzeit.edu/courtjudgments/ShowDoc.aspx?ID=53795.

8 Judgement No. 533/2018, Jerusalem Court of Appeals, 30 September 2018.

9 Judgement No. 284/2017, Ramallah Court of Appeals, 25 May 2017.

10 Judgement No. 92 of 2008, Palestinian Court of Cassation, (Five-judge panel), 29 June 2009, available at AL-MUQTAFI: http://muqtafi.birzeit.edu/courtjudgments/ShowDoc.aspx?ID=53795; Judgement No. 423 of 2009, Palestinian Court of Cassation, (Five-judge panel), 24 January 2009, available at AL-MUQTAFI: http://muqtafi.birzeit.edu/courtjudgments/ShowDoc.aspx?ID=60076.

11 Court of Cassation, Civil Judgements, Objection No. 2407 of 1998, p. 3181.

12 Judgement No. 92 of 2008, Palestinian Court of Cassation, (Five-judge panel), 29 June 2009, available at AL-MUQTAFI: http://muqtafi.birzeit.edu/courtjudgments/ShowDoc.aspx?ID=53795.

13 Judgement No. 1309/2019, Jerusalem Court of Appeals, 23 January 2020.

14 Judgement No. 511 of 2010, Palestinian Court of Cassation, 11 January 2011, cited in Nassrah, Ahmed. *The Palestinian Labour Law*. n. p., 2009, p. 222; Judgement No. 423 of 2009, Palestinian Court of Cassation, (Five-judge panel), 24 January 2009, available at AL-MUQTAFI: http://muqtafi.birzeit.edu/courtjudgments/ShowDoc.aspx?ID=60076; Judgement No. 265 of 2008, Palestinian Court of Cassation, (Five-judge panel), 7 April 2009, available at AL-MUQTAFI: http://muqtafi.birzeit.edu/courtjudgments/ShowDoc.aspx?ID=53764.

15 Court of Cassation, Civil Judgements, Objection No. 284 of 1998, p. 2119.

16 Court of Cassation, Civil Judgements, Objection No. 5894 of Judicial Year 75, 5 February 2006, Technical Office 57, p. 113.

17 Judgement No. 251/2017, Ramallah Court of Appeals, 17 April 2017.

18 Judgement No. 264/2016, Jerusalem Court of Appeals, 15 June 2016.

19 Court of Cassation, Civil Judgements, Objection No. 26 of Judicial Year 40, 27 December 1975, Technical Office 26, Vol. 2, p. 1699.

20 Court of Cassation, Civil Judgements, Objection No. 443 of Judicial Year 34, 4 February 1970, Technical Office 21, Vol. 1, p. 246.

21 see, Article 28 of the Kuwaiti Labour Law No. 6 of 2010; Objections 66 and 109/2001, Labour Cassation, Session of 20 May 2002; Objections 200 and 205/2006, Labour Cassation, Session of 2 June 2009; Objection 234/2006, labour Cassation, 8 June 2006. See Salah al-Jasim Systems of Legal Principles and Judicial Precedents.

22 Article 50, Kuwaiti Labour Law.

23 Court of Cassation, Civil Judgements, Objection No. 333 of Judicial Year 31, 12 May 1965, Technical Office 16, Vol. 2, p. 564.

24 Court of Cassation, Civil Judgements, Objection No. 1947 of 1998, 5 December 1998, p. 1260.

25 Court of Cassation, Civil Judgements, Objection No. 3094 of 2004, 15 February 2005, p. 1728.

26 According to the judgement on objection for cassation: “Pay for work. The principle is pay for work. (If the worker does not perform a work during a particular period of time, he is not entitled to be paied for that period). Objections No. 793 and 789 of 2014, Labour Case 1, Session 1, May 2017, Kuwaiti Court of Cassation.

27 Judgement No. 92 of 2008, Palestinian Court of Cassation, (Five-judge panel), 29 June 2009, available at AL-MUQTAFI: http://muqtafi.birzeit.edu/courtjudgments/ShowDoc.aspx?ID=53795.
